# Nine Months With a Pin: Thoracotomy After Repeated Bronchoscopic Failures in a 16-Year-Old With Hijab Syndrome

**DOI:** 10.7759/cureus.100937

**Published:** 2026-01-06

**Authors:** Saad Eddine J El Hariri, Tarek M Omais, Dima Mokhadder, Bahaa Bou Dargham

**Affiliations:** 1 Faculty of Medicine, Beirut Arab University, Beirut, LBN; 2 Department of Surgery, Hammoud Hospital University Medical Center (HHUMC), Saida, LBN; 3 Department of Anesthesia, Hammoud Hospital University Medical Center (HHUMC), Saida, LBN

**Keywords:** adolescent thoracotomy, bronchial foreign body, bronchoscopy failure, foreign body inhalation, hijab syndrome

## Abstract

Inhaled sharp foreign bodies, such as pins, are commonly managed through bronchoscopic retrieval. However, challenges arise when such efforts fail, requiring escalation to surgery. We report a rare case of a 16-year-old female who presented nine months after inhaling a pin. During this period, multiple bronchoscopic removal attempts were unsuccessful. Imaging localized the foreign body to the middle lobe of the right lung. Given the chronicity of the case and endoscopic failure, the patient underwent thoracotomy, through which the pin was retrieved. The postoperative course was uneventful, and she was discharged without complications. This case underscores the importance of recognizing the limitations of bronchoscopy and the need for timely transition to surgery. Establishing clear criteria for escalation to thoracotomy is essential to optimize outcomes and ensure effective communication with patients and families.

## Introduction

Foreign body aspiration (FBA) is a leading cause of death in children, particularly those under five years of age. FBA is defined as a partial or complete obstruction of the airway by a solid or semi-solid object. Clinical presentation varies and may include gagging, coughing, stridor (a high-pitched breathing sound due to airway obstruction), wheezing, or impaired oxygenation. Severe complications include hypoxic-ischemic brain injury, neurological damage caused by inadequate oxygen delivery, and pulmonary hemorrhage. If not promptly diagnosed and managed, it may lead to significant complications, including chronic cough, recurrent infections, or, rarely, lung abscess or pneumothorax [1]. A recently recognized pattern of FBA, termed "hijab syndrome," predominantly affects young females and results from pinning the headscarf in place while adjusting it [2].

## Case presentation

**Figure 1:**
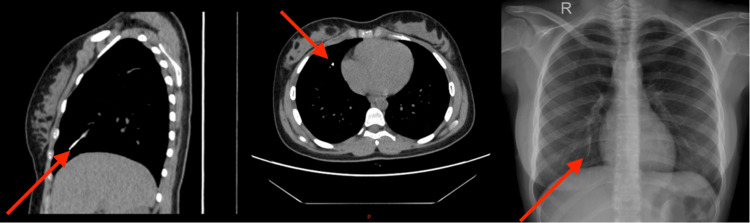
Preoperative imaging showing a metallic linear foreign body (2.6 cm) in the distal anterolateral segment of the right middle lobe bronchus

**Figure 2:**
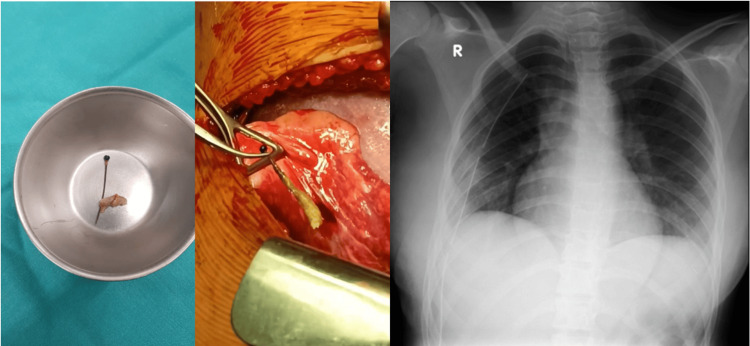
Intraoperative and postoperative findings showing the retrieved hijab pin successfully removed via thoracotomy

**Figure 3:**
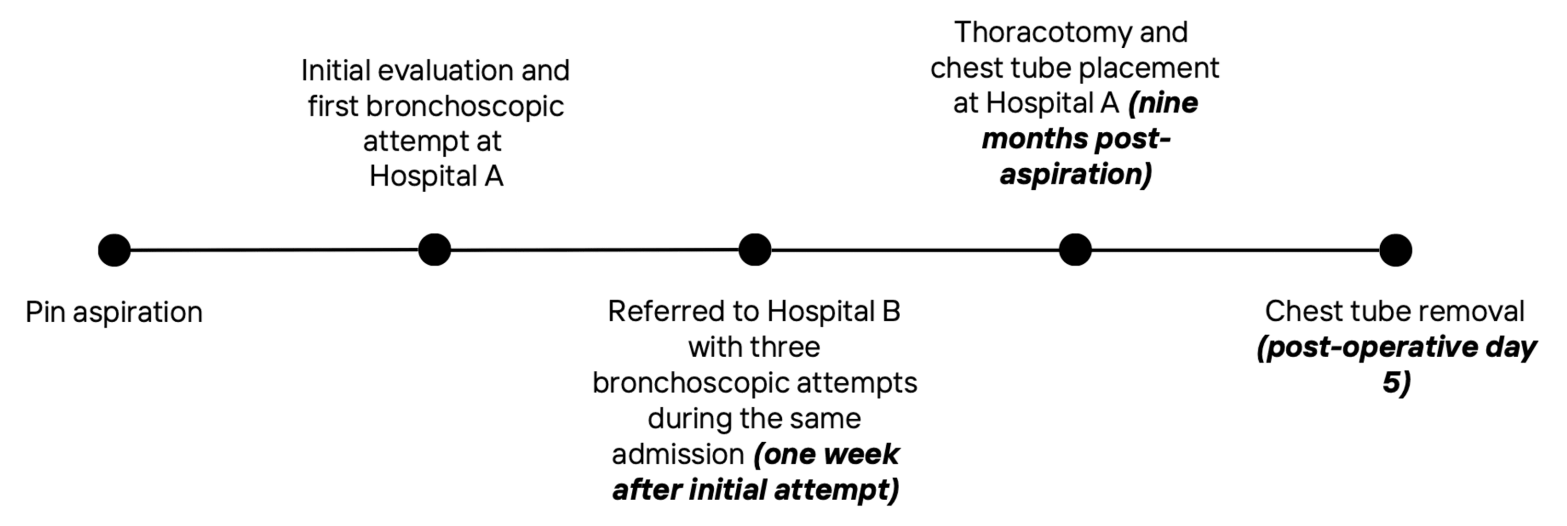
Timeline of clinical events following pin aspiration

A 16-year-old, previously healthy, veiled female presented for elective surgical removal of a headscarf pin, nine months after accidental aspiration. The pin, which she had been holding in her mouth, was inhaled during a fall while adjusting her headscarf. She subsequently developed a persistent dry cough and dyspnea. Imaging confirmed a metallic foreign body in the right middle lobe. Initial and subsequent bronchoscopic attempts at two institutions were unsuccessful. Due to financial limitations, no further intervention was pursued until nine months later, when she returned with exertional dyspnea and intermittent right-sided chest pain. She denied fever, nausea, vomiting, or other systemic symptoms. A follow-up CT scan confirmed the persistence of the pin. The patient was admitted for elective thoracotomy. Preoperative labs, chest CT, and chest X-ray were done (Figure 1). A right posterolateral thoracotomy through the fifth intercostal space revealed the pin embedded in the lung parenchyma. It was dissected and removed with minimal trauma. Hemostasis was secured, one chest tube was placed, and the thoracotomy was closed in layers. Postoperative recovery was uneventful. The chest tube was removed on postoperative day five, and she was discharged in stable condition (Figure 2). The full timeline of clinical events is summarized in Figure 3.

## Discussion

Tracheobronchial FBA is a common cause of morbidity and mortality in children, with asphyxia being a leading cause of death in severe cases [3]. In recent years, a high-risk subgroup has been females who inhale headscarf pins, a pattern referred to as “hijab syndrome” [2]. This results from the habit of holding the pin in the mouth while securing the headscarf. Any abrupt action, such as deep inhalation, talking, coughing, or laughing, may lead to aspiration. Cough and dyspnea are the most frequently reported symptoms [3]. Diagnosis relies on a thorough history and chest X-ray demonstrating the radiopaque pin. CT scanning offers precise localization and orientation, as in our case. In the present case, the foreign body remained in situ for nine months despite four bronchoscopic attempts at two institutions within the first weeks after aspiration, highlighting the risk of chronic retention when early endoscopic retrieval fails. While FBA in children occurs equally in both bronchi, headscarf pins are more often found in the left bronchus due to Bernoulli’s phenomenon, whereby negative pressure during coughing is greater on the narrower left side. Rizk et al. reported 53% of cases on the left [4].

In contrast, our patient had the pin lodged in the right bronchus, which is notable as a variation. Bronchoscopy has markedly reduced the need for surgery in pin aspiration [5]. Rigid bronchoscopy, preferred in children, allows better airway control and suctioning. During rigid bronchoscopy, the pointed end should be grasped and withdrawn into the bronchoscope to avoid mucosal injury. Flexible bronchoscopy may be preferred in adults, especially for distal airway foreign bodies [5]. When bronchoscopic retrieval fails, surgical intervention becomes necessary. In selected cases, video-assisted thoracoscopic surgery (VATS) has been described as a less invasive alternative to thoracotomy and can also be used in such cases of FBA [6]. Rizk et al. and Baram et al. reported thoracotomy rates of 6-7% due to failed bronchoscopic retrieval [2,4]. In our case, the pin remained lodged for nine months despite four bronchoscopic attempts, eventually necessitating thoracotomy. This highlights the importance of early diagnosis and timely escalation to surgery when endoscopic approaches fail. Although objective postoperative pulmonary function tests and oxygen saturation trends were not systematically recorded due to the retrospective nature of this case, the patient demonstrated clear clinical improvement with resolution of dyspnea and chest pain during follow-up.

Written informed consent for publication of clinical details and images was obtained from the patient’s legal guardian, in accordance with institutional and journal requirements.

## Conclusions

This case illustrates the challenges of scarf pin aspiration in young females who hold pins in their mouths while adjusting headscarves. While bronchoscopy is the first-line approach, repeated failure should prompt consideration of intervention, and both VATS and thoracotomy should be kept in mind. Emphasizing preventive education to discourage the habit of holding pins in the mouth is crucial to reducing complications and preventing similar aspiration events in the future. Clinicians should maintain a high index of suspicion in symptomatic patients and consider early imaging and intervention to optimize outcomes.
